# Profile: Professor Ian Jones

**DOI:** 10.1192/pb.bp.116.054296

**Published:** 2016-08

**Authors:** Julia Bland

Bespectacled Professor Ian Jones is an unpretentious, fluent and animated man with a dazzling set of credentials, including over 18 500 citations of his research output.

His Chair, at Cardiff University School of Medicine, in the Department of Psychological Medicine and Clinical Neuroscience, is in Perinatal Psychiatry. He is the director of the National Centre for Mental Health (NCMH), and runs the Bipolar Disorder Research Network (BDRN). In 2013, he won the Royal College of Psychiatrists' Academic Researcher of the Year Award.

What I immediately liked about him was his pragmatic determination to improve the quality of clinical performance by whatever means comes to hand. Ian Jones does not see himself as a purist researcher: ‘The ultimate goal of research is making things better for people with these conditions … via the development of services and stigma reduction. I'm primarily a clinician rather than a scientist’.

His first exposure to research was doing an MSc in Cardiff as a psychiatric trainee, subsequently a Wellcome training fellowship in Birmingham, and a year in the USA, all looking at bipolar disorder and postnatal issues, before settling in Cardiff. He is a Welshman from the Rhondda Valleys. His grandfather had to leave school aged 15 to go down the mines because his father had died in a mining accident. Jones' own father was a schoolteacher who became a Minister, and the family moved to England when Ian Jones was a child. He remains grateful for the opportunity he had to get a medical education without student debt, and fears for the potential narrowness of medical school intake in these harsher times. He now lives in Worcester with his GP wife, so he is well attuned to the difficulty of accessing secondary psychiatric care. They have three children between the ages of 13 and 19 and he is a fan of modern British folk music and the Worcester Warriors (rugby, of course, not football).

While not wishing to be too politically provocative, he admits that leaving the NCMH deliberately ambiguous (Wales, the nation or the whole UK?) is a tacit challenge to all the London institutions which do not hesitate to call themselves ‘national’. On the other hand, he fully acknowledges that they are funded by Welsh healthcare research money.

Professor Nick Craddock has been a career-long mentor, a role Jones describes as ‘particularly important for clinical academics’.

Jones appreciates the particular opportunities being a clinical academic affords: ‘you still treat patients but you have a bit more flexibility than in a pure NHS job and you get to do research as well’. In his role as a researcher it seems likely that his affable personality lends itself to collaborative research projects (e.g. as part of the Wellcome Trust genome-wide association study group) and his enthusiasm for patient involvement is not just as a politically correct add-on but key to the success of ventures such as the BDRN, to which he is utterly committed.

## People, not patients

Jones' attitude of respect and interest in his patients comes across clearly. Having met him, it is easy to imagine how thoughtful he would be in the clinical encounter. Not adopted self-consciously as a correct attitude, but genuinely felt. This sits congruently with his attitude to our clumsy, inaccurate (and proliferating) diagnostic categories. He is not anti diagnosis, and points out how helpful diagnosis can be in directing people to the treatment and support they need. But with hundreds of diagnostic categories and subcategories in DSM-5, he thinks most practising clinicians think they could do with ‘about 15’.

## Science

Jones has contributed to important scientific advance, particularly in the area of the clinical and molecular genetics of bipolar disorder and puerperal psychosis. His first big Wellcome-Trust-funded research study was of bipolar twin pairs, where he heard from the patients directly about their severe episodes of postpartum illness and noticed how often this was the first episode of continuing bipolar disorder. He is still excited by the many unanswered questions raised by the link between childbirth and serious mental illness, and wonders whether this link may be a clue to understanding the aetiology of mood disorders in general.

The research questions that preoccupy him currently are ‘What is the importance of biological/hormonal triggers and immunological factors?’ and ‘How does sleep disruption or change in circadian rhythms play into the aetiology of mental illness?’. In collaboration with Professor Lisa Jones and the University of Worcester, he is part of a current, prospective study of high-risk women with a history of bipolar disorder or puerperal psychosis, asking them to wear ‘actigraph’ watches to monitor their sleep. The plan is to determine what factors increase the risk of a severe postpartum recurrence, such as the link between sleep disturbance and prodromal symptoms: is sleep disturbance a trigger or/and a symptom of illness?

He is also involved in a joint study with the University of Worcester and Oxford University using True Colours (oxfordhealth.truecolours.nhs.uk), a web-based system for monitoring mood. What he hopes will emerge from this work is a more subtle conceptualisation of mood variation in lived experience. People with bipolar disorder are ‘not just euthymic with episodes of mania or depression … it's more complicated and variable than that.’

The risks of postpartum psychosis in mood disorders is high: in a study reported in 2013, with a sample of nearly 2000 women with bipolar disorder or recurrent major depression, more than 66% had at least one episode of perinatal mood disorder and around 20% of women with bipolar disorder had experienced postpartum psychosis.^1^ In 2001, in the *American Journal of Psychiatry*, Jones and others reported the heavy genetic loading in puerperal psychosis, finding evidence that the puerperal trigger in bipolar disorder was familial and suggested that the majority of postpartum psychotic episodes can be viewed as bipolar disorder with a puerperal trigger.^2^ While 26% of all deliveries in women with bipolar disorder lead to puerperal psychosis, a huge 74% of women with bipolar disorder and a family history of puerperal psychosis in a first-degree relative will have a puerperal psychosis compared with 38% without a family history.^2^

With Professor Nick Craddock and Professor Lisa Jones at the University of Worcester, he leads the Bipolar Disorder Research Network (BDRN, www.BDRN.org), which has recruited over 6000 people with bipolar disorder into research across the UK. The BDRN sample is a large contribution to the psychiatric genomics consortium bipolar work and he is excited about the way genetics is improving our understanding of mental illness. For example, the genetic overlap in the risk of bipolar disorder, recurrent major depression and schizophrenia,^3^ which obviously has major implications. ‘The point is that the diagnostic complexity doesn't reflect the underlying scientific understanding’. No wonder so many of our patients with chronic illness run the gamut of diagnoses over their psychiatric ‘career’.

With bipolar disorder, Jones thinks it is both under- and over-diagnosed. For some people, the diagnosis provides access to much needed and overdue specialist help. For others, it may be a way of avoiding the less palatable diagnoses, such as personality disorder.

## Post-partum illness

Jones' commitment to this complex field is unequivocal:

‘The postpartum period is so important … it's a devastating time to be unwell. Women with postpartum psychosis need high-quality in-patient treatment with pharmacology initially and then talking treatments to come to terms with what has happened to them, and the guilt and so on … ’.

There is no mother and baby unit in Wales now and Jones deplores the postcode lottery of service availability, which he, the Maternal Mental Health Alliance and Action on Postpartum Psychosis are seeking to change. Perhaps the new investment in mental health announced recently will improve this area of psychiatric care?^4^

## Public education

For Jones there is a seamless continuity between destigmatisation, public education and the involvement of people with lived experience of illness in research. He told me he was struck by a figure quoted that ‘only a third of cancer patients are involved in research’, with the implication that in medicine the expectation of patient involvement in research is vastly higher than it is in psychiatry.

In contrast to a ‘paternalistic, protective’ attitude (which he sees as having been prevalent in psychiatric services hitherto), he wants people to have an opportunity to participate, with the carrot of benefiting others in the future. Recruitment into the BDRN has been much easier than he anticipated: ‘we said we'd recruit 6000 but didn't think it would be easy … but we have … and collaboration with Bipolar UK has been key, one third of our recruits have come from them’. The NCMH has also recruited over 6000 people.

‘Interestingly, the third sector and people with lived experience are not difficult to win into cooperation: they see the need. It's the mental health services themselves who are harder to engage’.

Another patient research initiative he mentions is the True Colours project he is involved in with Oxford University, where people can monitor their mood over time: ‘this is reconceptualising mood disorders and we should be doing prospective studies’. He is frustrated by the stigmatising views of some ethics committees who are reluctant to see psychiatric patients as being capable of deciding for themselves to participate in research. Demonstrating the only-too-familiar lack of parity between mental and physical health, committees can believe that ‘decisions have to made on behalf of psychiatric patients, who need protection’. He quotes the outrageous differential against mental health in research funding, amply demonstrated by the fact that, for each £1 of government spending on cancer research, £2.98 is raised from the charity sector, compared with only 0.01 pence per government pound on mental health research.^5^

In the NCMH he wants to promote research integrated into the NHS, as part of its role, of ‘what we do’. His appetite for public education has led him to some interesting places. As script advisor for the British TV soap opera *EastEnders*, he helped with the storyline of Stacey Branning, whose descent into puerperal psychosis was viewed by over 9 million people.^6^ He has been interviewed on BBC Radio 4's *Woman's Hour*, and encouraged the YouTube video of a famous 64-year-old Welsh Rugby referee, Clive Norling, impressively describing his severe depressive episode. He has helped instigate the ‘Bipolar Education Programme Cymru’, a direct mixture of group sessions and an online interactive module in Wales, and chaired and is still a trustee of Action on Postpartum Psychosis.

## ‘Muscular psychiatry’

In a controversial letter to the *British Journal of Psychiatry* in 2008,^7^ he and Nick Craddock called for reclaiming the medical core in psychiatry and warning against a ‘down-grading’ of the medical aspect of the role in favour of psychosocial care. It was a call to arms, asserting that scientific rigour and patient involvement go hand in hand, valuing an unsentimental, rigorous, ‘muscular’ psychiatry.

Jones describes the unhelpful diffidence of the specialty, its reluctance to assert its own value: ‘We are very nice, reasonable people who don't put forward our agenda as strongly as we should … we need to stand up more for the role doctors play’. He affirms our unique capacity to participate in the full range of what patients need: a biological perspective and medication, talking treatments, and educating patients and the public about mental health. He urges the profession ‘not to play into therapeutic pessimism … sure there are side-effects of medication, and we need better, safer drugs, but drugs work in psychiatry … as well or better than in many physical health conditions’.

Having worked in general medicine before becoming a psychiatrist, Jones sees the psychiatrist as an effective prescriber, who should ‘stand up for medicine’ while not losing the breadth of perspective that encompasses the social and psychological as well as the pharmacological.

## Recruitment

Jones sees recruitment as the most important challenge facing the Royal College of Psychiatrists: ‘We have to get the brightest and best students to come into psychiatry’. He proposes an early intervention, perhaps at the sixth-former stage: ‘We could say to [the sixth-formers]: “If you are considering psychology, sociology, if you are interested in the mind/brain stuff, why not do psychiatry via medicine?” ’

This idea reminded me of a conversation relayed to me between a friend and her three adult daughters in their late 20s, one lawyer, two accountants. They are all fed up and wish they could be in the psychiatry/psychology field. As my friend said: ‘These jobs are about what makes people tick … in the end, it's the only thing that matters, or is interesting’. Ian Jones' own experience of psychiatry teaching at St George's medical school was inspiring, ‘the best delivered teaching block’, and he rightly emphasises the importance of exposing students to the excitement and to hearing the lived experience of patients, the ‘how psychiatry saved my life’.

We know medical students are exposed to the disparaging, prejudiced remarks of other doctors about psychiatry. ‘You are too good to be a psychiatrist’, Jones was told. The attitude of medical students to psychiatry as ‘not proper medicine’ has been directly captured in focus groups at Durham (personal communication, S. Sinclair, 2014). Jones is frustrated by therapeutic pessimism about psychiatry:

‘The idea that no-one ever gets better … it's just not true … we get far better outcomes than in chest medicine where I worked before becoming a psychiatrist … We must be more positive about ourselves. What medics bring to the table is often derided, as if using psychotropic drugs is by default, or a sign of, failure. This needs to be robustly challenged.’

‘In a survey of women who reflected on their experience of post-partum psychosis, they were very clear that it was admission and drug treatment that they needed initially. They needed secondary psychological treatment when they had recovered from the acute illness, to deal with the guilt and so on, but they valued medical care … ’.

He makes a cogent plea for psychiatry as uniquely holistic:

‘This is how we help people, this is the case we need to make. In perinatal psychiatry, particularly, we cannot ignore all aspects of treatment. You cannot be a hardened psychopharmacologist or a pure psychotherapist’.

He becomes increasingly passionate about the integrated assessing, prescribing, listening roles of the psychiatrist: ‘Giving hope to people is the most important thing we do, letting people tell their story is validating, giving them hope that they can get better’. He is concerned that as biological understanding improves, medicine may encroach on our terrain (e.g. paediatricians taking over attention-deficit hyperactivity disorder) and the danger of handing over responsibility for conditions is the danger of losing our unique and invaluable holistic approach. Conversely, he does not see psychiatry as ‘all about wishy-washy feelings. There is lots of interesting science to be done at interface of neuroscience and mind … where the most exciting stuff of next 40 years will be unfolding,’ he predicts.

As if he knows he is getting a bit carried away on a wave of positivity which may not strike a chord with a hard-pressed CMHT consultant, he admits it is hard to maintain positivity in underfunded services, and he knows it is easier for an academic. But Ian Jones is not twiddling his thumbs, and his energy and optimism seem like a refreshing tonic in the jaded grey days of February.
